# Genome wide association studies in yam reveal the challenge of high heterozygosity

**DOI:** 10.1038/s41598-025-10344-z

**Published:** 2025-07-24

**Authors:** Paterne A. Agre, Kwabena Darkwa, Iseki Kohtaro, Ryo Matsumoto, Asrat Asfaw

**Affiliations:** 1 International Institute of Tropical Agriculture, Ibadan, 200001 Nigeria; 2 Savanah Agricultural Research Institute, CSIR-SARI, Tamale, Ghana; 3 Japan International Research Center for Agricultural Sciences, Tsukuba, Japan

**Keywords:** Yam, Mapping, Yield, Heterozygote level, Prediction, Biological techniques, Genetics, Plant sciences

## Abstract

Yam (Dioscorea spp.) is a herbaceous vine crop valued for its starchy tubers, which are rich in essential nutrients. Its genome is highly heterozygous, contributing to considerable genetic diversity and adaptability. Understanding the polymorphism information content (PIC) of genetic markers is critical for enhancing key agronomic traits such as yield. In this study, we conducted a genome-wide association analysis that accounts for heterozygosity to investigate fresh tuber yield variation in white Guinea yam (Dioscorea rotundata Poir). A total of 173 genotypes including 86 elite breeding clones, 77 genebank accessions, and 10 farmer varieteies were genotyped through whole-genome resequencing, yielding approximately 1.6 million single nucleotide polymorphism (SNP) markers. Association analysis was performed using a multi-locus mixed linear model (MLM), incorporating kinship matrices derived from marker subsets grouped by PIC levels (≤ 0.1, 0.1–0.2, 0.2–0.4, and > 0.4), alongside population structure. The analysis revealed that high-PIC markers had greater influence on trait associations. Twelve stable SNPs were significantly associated with fresh tuber yield. Functional annotation of these markers revealed putative genes related to plant growth and cellular regulation. Notably, markers located in heterozygosity-rich genomic regions were linked to high-yielding genotypes, while those in homozygous regions were associated with lower yields. These findings underscore the potential of PIC-based marker selection and highlight the value of integrating heterozygosity metrics into genomic-assisted breeding strategies for improving fresh tuber yield in white Guinea yam.

## Introduction

Yam (*Dioscorea* spp.) is a staple crop, significantly contributing to the dietary needs of millions across Africa, the Pacific and the Caribbean^1–3^. This crop is an especially valued food source that is highly socio-culturally relevant in Africa^4^. The current global production of yam stands at approximately 88 million metric tons, of which Africa accounts for about 98%^5^. Its achieved yield is on average around 8.5 tons per hectare, which is well below its potential yield of 50 tons per hectare^6^. Addressing this yield gap with state-of-the-art genetic improvement that employs effective, efficient and precise methods is imperative to enhance food security, particularly in the face of climate change and population growth challenges. Classical breeding programs for yam are impeded by crop’s complex genome, long breeding cycles, and high heterozygosity^7^. Genome-wide association study (GWAS) is among the powerful tools that revolutionized our understanding of the genetic basis of complex traits in various organisms and help to advance breeding efforts by facilitating the development of improved varieties with greater efficiency^8^. GWAS studies leverage on the natural variation in populations to identify genetic loci associated with phenotypic traits, offering insights into the genetic architecture underlying these traits^9^. However, highly heterozygous plant species such as yam, wherein individual plants harbor different alleles at a given locus, significantly influence the power of GWAS, both in terms of the accurate detection of the relevant genetic markers associated with key yam traits and interpreting the underlying genetic mechanism^7,10,11^. In populations with extensive heterozygosity, disentangling the effects of individual genetic variants on traits is challenging, particularly if epistatic interactions (alleles potentially interacting in non-additive ways) are prevalent^12^. This is particularly important for complex traits such as yield, which is highly influenced by multiple genetic factors^13^. Yield in yam is a complex trait influenced by numerous small-effect variants^14^. This inherent complexity necessitates refined methodology within GWAS to detect the trait-linked markers accurately. A series of studies have shed light on the potential of GWAS in the identification of genetic variants linked to key traits in yam^15–18^. However, none of the previous studies critically looked at the influence of heterozygosity on GWAS accuracy.

Given the challenge of correctly modeling the relationship between phenotype and genotype with GWAS, especially when heterozygosity is prevalent in the genome, it is crucial to design studies that maximize their potential to uncover meaningful genetic associations^20^. One approach is to stratify the population based on levels of heterozygosity, allowing for more targeted analyses that consider the genetic background of individuals. This stratification can help identify subsets where genetic effects are more easily detectable, reducing the noise introduced by complex genetic interactions^21^. Additionally, genotype imputation, population selection, Single Nucleotide Polymorphism (SNP) selection, and multi-population are the common strategies used to address the impact of heterozygosity during the GWAS analysis^22,23^.

In this study, we used SNP selection focusing on SNPs with varying levels of heterozygosity and employed a Mixed linear model (MLM) to perform trait association analysis. MLMs account for population structure and relatedness by incorporating kinship matrices derived from genetic markers. Heterozygosity influences these models, and using heterozygosity-based kinship matrices can improve their accuracy^23^. The main objective of this study is to assess the impact of the heterozygosity on the GWAS accuracy and the genome prediction for a complex trait like tuber yield in yam.

## Results

### Variation in fresh tuber yield per plant

The analysis of variance revealed highly significant differences (*p* < 0.01) among the genotypes, year and genotype by year interaction effect for tuber yield per plant (Table 1). Averaged across the year, tuber yield per plant varied from 0.117 to 2.617 with a mean of 1.05 kg plant^-1^ (Supplementary Fig. 1). Broad sense heritability for tuber weight per plant was high (0.66).

**Table 1:** Mean square and heritability estimate for tuber yield per plant in the association panel evaluated across 2 years.

Trait	Mean squares			Error means square	Mean	Range	H^2^
	Genotype (G)	Year (S)	G × S				
Tuber yield per plant (kg)	0.56**	3.00**	0.26**	0.19	1.05	2.23	0.66

H^2^: Broad sense heritability, G x S: genotype by year interaction effect, **: significant at 1% probability level.

### Summary statistics for marker data

Of the 136,000 SNP markers retained after filtering, the expected heterozygosity varied from 0.1 to 0.5, while the observed heterozygosity varied from 0.1 to 0.457. The Minor allele frequency ranged from 0.05 to 0.5, and the polymorphism information content varied from approximately 0.1 to 0.42, with 0.28 as an average. Markers are unequally distributed across the 20 yam chromosomes (Supplementary Table 1). The number of SNP markers per chromosome varies from 1825 to 13,992, with the highest density on chromosome 9 (Supplementary Fig. 2).

### Genetic diversity and population structure

**Fig. 1:**
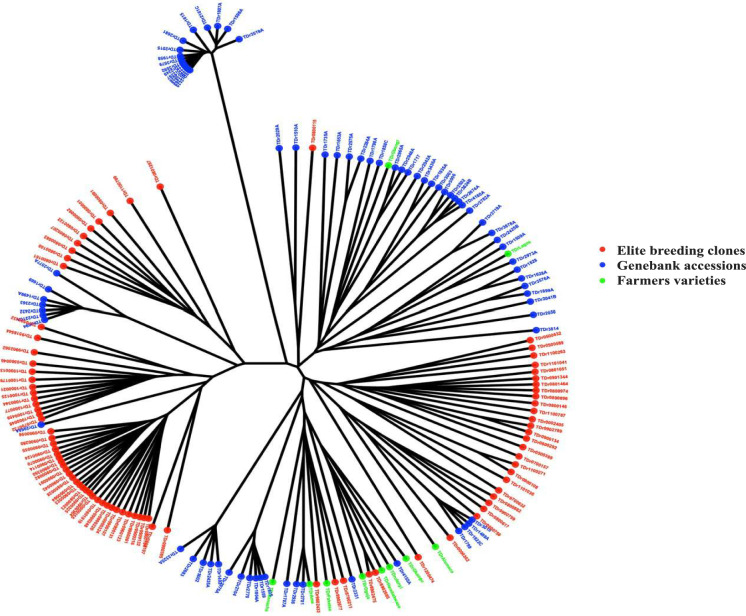
Phylogeny tree-based IBS matrix displaying the relationship among elite breeding clones, genebank accessions, and farmers’ varieties.

The genetic distances ranged from 0.06 to 0.36, and the highest genetic distance was reported between elite breeding clones and farmers varieties. The phylogenetic analysis of 174 *Dioscorea rotundata* accessions revealed clear clustering patterns corresponding to elite breeding clones, genebank accessions, and farmers’ varieties. Elite clones (red) were primarily grouped into two main clusters, indicating limited genetic divergence within this group. In contrast, genebank accessions (blue) showed a wider distribution across the tree, reflecting a higher level of genetic diversity. Farmers’ varieties (green) were distributed across several branches, occupying intermediate positions between elite clones and genebank accessions. A few accessions from different categories clustered closely together, suggesting potential genetic overlap among groups. Notably, the root of the tree was primarily formed by genebank accessions (Fig. 1).

**Fig. 2:**
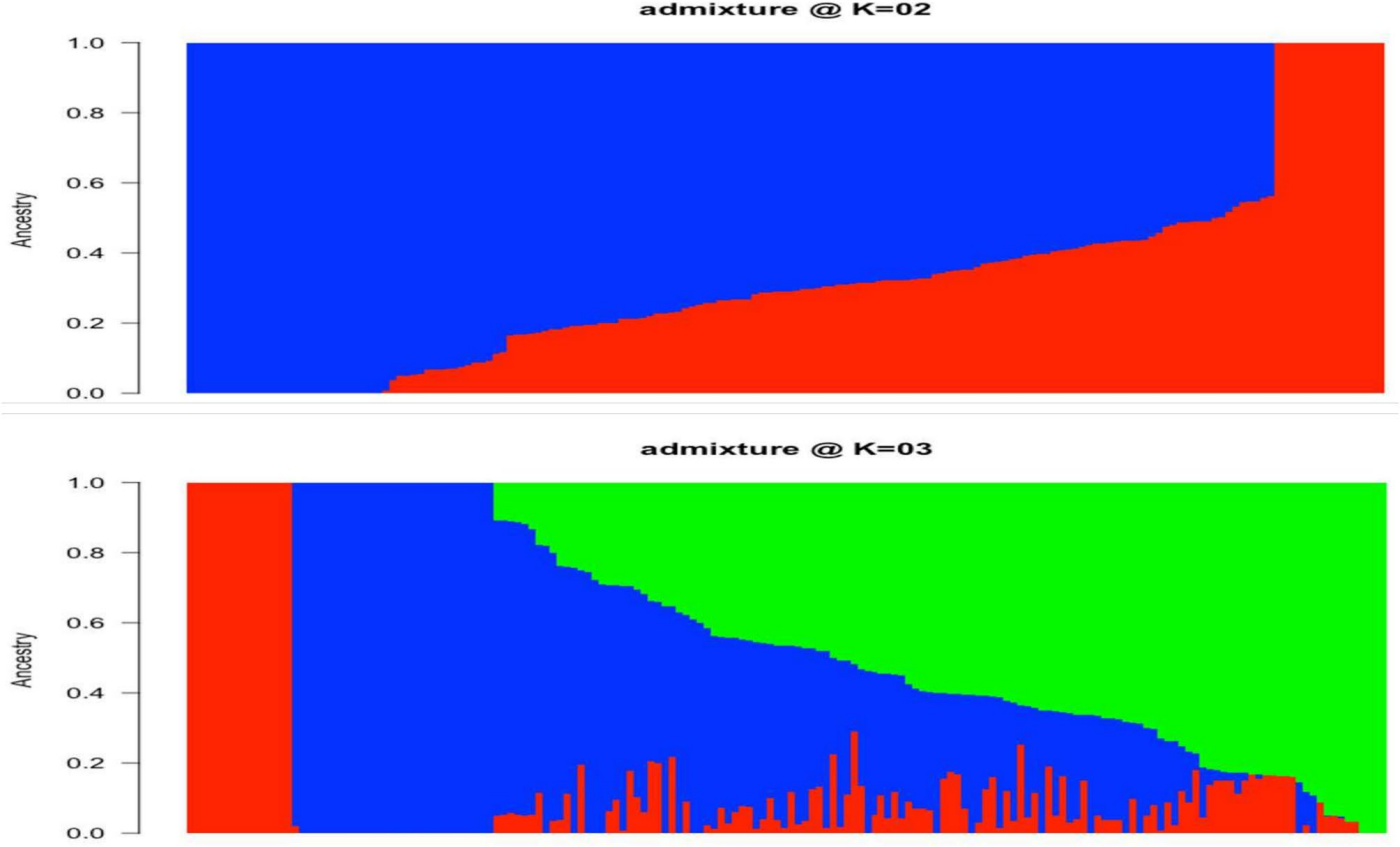
Population structure at K = 2 (a) and 3 (b). The color is associated with each cluster, and each bar represents the genotype.

Population structure analysis indicated potential grouping at k = 2 and k = 3 using the Bayesian Information Criteria (BIC) (Fig. 2). With a probability ancestry threshold of 70%, 157 genotypes were successfully assigned to three subpopulations at k = 3. The remaining genotypes, which had an ancestry probability below 70%, were considered admixed, reflecting genetic contributions from multiple subpopulations. At k = 3, subpopulation 1 (red) consisted of farmers varieties with no admixed genotypes. Subpopulation 2 (blue) included genebank accession, among which some genotypes had an ancestry probability of less than 70% and were considered as admixt. Subpopulation 3 (green) comprised of elite breeding clones and displayed the highest level of admixture (Fig. 2).

### Effect of markers polymorphism on the population structure

**Fig. 3:**
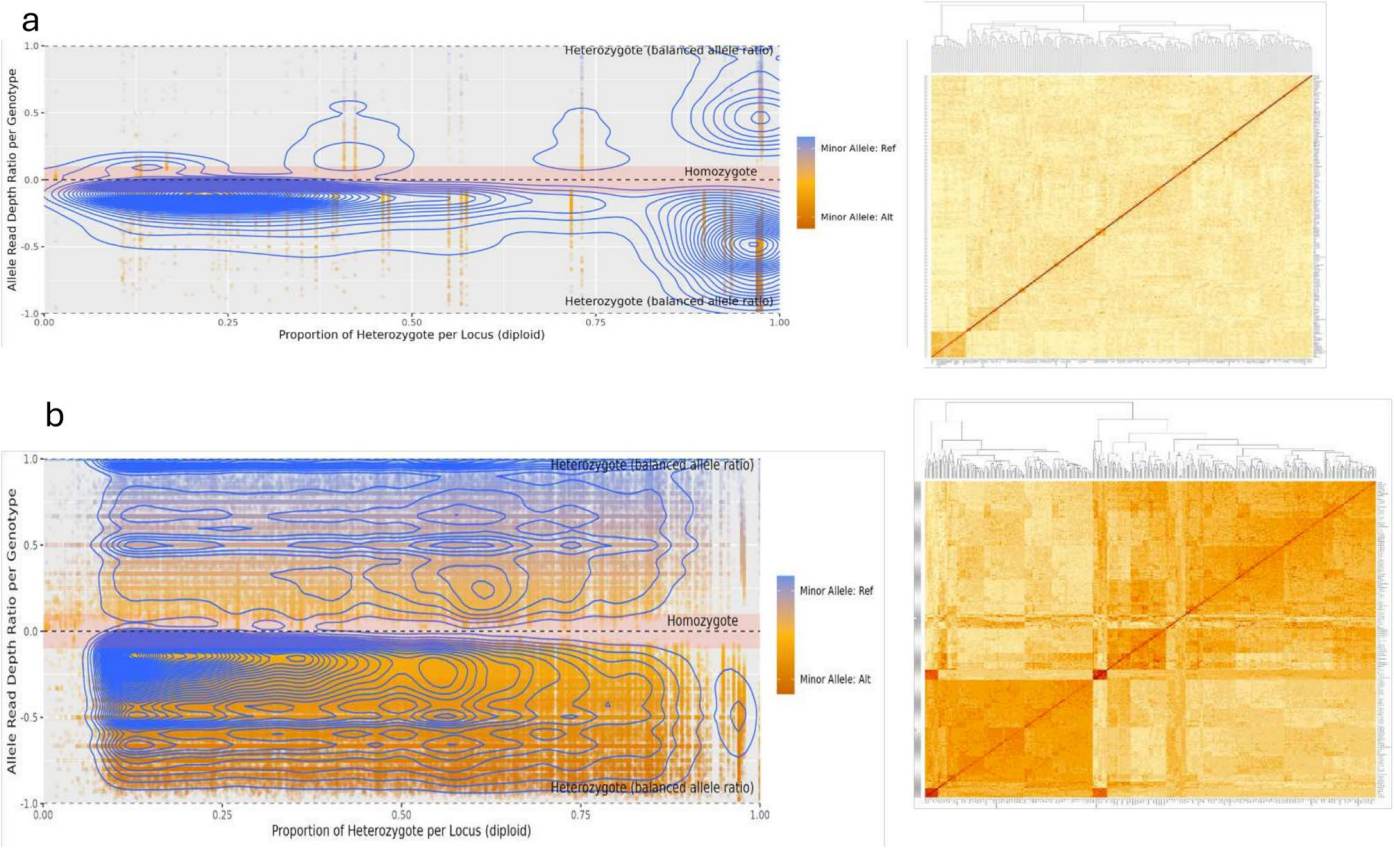
Heatmap clustering based PIC. a: Population stratification using markers with low PIC < 0.2 (no clear population structure) and b: Population stratification using markers with high polymorphism information > 0. 2 content (clear population structure observed).

The population structure-based polymorphism level revealed a high impact on the population stratification (Fig. 3). Makers with low PIC displayed inadequate or inability to cluster properly compared to makers with high PIC (Figs. 3a). Using markers with a high PIC level, the 173 genotypes were grouped in three well-defined clusters (Fig. 3b).

### Trait-association for tuber yield per plant

We investigated the association between genetic markers and a specific trait using six different methods (mrMLM, FASTmrEMM, ISIS EMBLASSO, pLARmEB, pKWmEB, and FASTmrMLM). We employed both a naive model and a kinship-based model (KQ model) to identify relevant Single Nucleotide Polymorphisms (SNPs).

- Naive Model

**Fig. 4:**
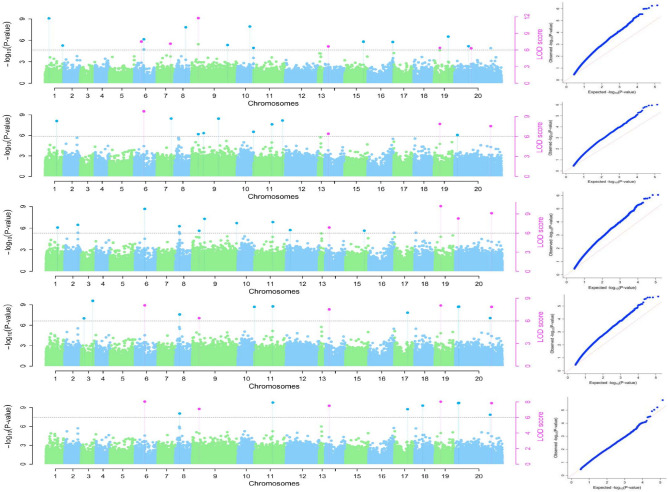
Manhattan and QQ plots of the tuber yield per plant using Naïve and K + Q models (The red and blue dots are for makers with significant LOD above the threshold.

In the naive model analysis, we identified 15 SNP markers distributed across 11 chromosomes (Fig. 4). These markers had high minor allele frequency (MAF) and high LOD scores but explained a low proportion of the total phenotypic variation (Table 2).

**Table 2:** Summary statistics of SNP markers link with the tuber yield per plant using different models and genetic methods.

Models	Methods	Markers	Chr	Pos	LOD	-log10(P)	r2 (%)	MAF	Alleles
Naïve	mrMLM	chrom_07_25584966	7	25,584,966	7.5914	8.4726	1.34	0.1433	C
		chrom_09_6543874	9	6,543,874	10.6343	11.5854	2.50	0.345	C
		chrom_15_18362333	15	18,362,333	7.4894	8.3678	1.23	0.3509	T
		chrom_19_9509263	19	9,509,263	6.1751	7.014	2.45	0.1228	T
		chrom_19_24000699	19	24,000,699	8.3917	9.2936	1.80	0.3889	T
		chrom_20_15694564	20	15,694,564	6.4184	7.2652	3.5	0.3041	T
	FASTmrMLM	chrom_01_4390222	1	4,390,222	11.6802	12.6509	1.28	0.1503	T
		chrom_01_28700972	1	28,700,972	6.7967	7.6552	0.23	0.2775	C
		chrom_06_8317403	6	8,317,403	8.8939	9.8078	Na	0.4653	A
		chrom_06_9260283	6	9,260,283	7.9063	8.7959	Na	0.2572	C
		chrom_09_21942129	9	21,942,129	6.8797	7.7407	Na	0.2254	A
		chrom_10_18940999	10	18,940,999	10.2145	11.1572	Na	0.3526	C
		chrom_19_9509263	19	9,509,263	16.736	17.7823	Na	0.1243	T
		chrom_20_13510828	20	13,510,828	6.676	7.5308	Na	0.4769	C
		chrom_20_15694564	20	15,694,564	6.1934	7.0329	Na	0.3064	T
	FASTmrEMMA	chrom_08_12524719	8	12,524,719	10.089	11.0291	12.0217	0.1618	T
		chrom_10_23363999	10	23,363,999	6.3631	7.2081	4.2781	0.1387	T
		chrom_16_23236778	16	23,236,778	7.4276	8.3043	5.3432	0.1358	G
	pLARmEB	chrom_07_25584966	7	25,584,966	6.6797	7.5346	Na	0.1416	C
		chrom_09_6543874	9	6,543,874	12.8199	13.8101	Na	0.3468	C
		chrom_14_4221541	14	4,221,541	6.1433	6.9811	Na	0.1156	G
		chrom_19_9509263	19	9,509,263	6.9529	7.816	Na	0.1243	T
		chrom_06_8317403	6	8,317,403	6.1267	6.964	Na	0.4649	A
		chrom_14_4221541	14	4,221,541	8.7141	9.6238	Na	0.117	G
		chrom_19_9509263	19	9,509,263	6.3294	7.1733		0.1228	T
	BLASSO	chrom_14_4221541	14	4,221,541	6.6444	7.4982	Na	0.1156	G
		chrom_19_9509263	19	9,509,263	6.368	7.2132	Na	0.1243	T
K1 + Q	mrMLM	chrom_07_26786068	7	26,786,068	8.6955	9.6047	Na	0.1462	A
		chrom_09_19610000	9	19,610,000	8.6816	9.5905	Na	0.1316	T
		chrom_10_23363999	10	23,363,999	6.7051	7.5608	Na	0.1374	T
		chrom_11_17979898	11	17,979,898	8.3993	9.3014	Na	0.4503	A
	FASTmrMLM	chrom_06_9260283	6	9,260,283	9.9477	10.8849	Na	0.2572	C
		chrom_11_11707748	11	11,707,748	7.8196	8.7069	Na	0.4335	T
		chrom_14_4221541	14	4,221,541	6.6959	7.5513	Na	0.1156	G
		chrom_19_9509263	19	9,509,263	8.8431	9.7558	Na	0.1243	T
		chrom_20_24056604	20	24,056,604	7.5576	8.4379	Na	0.3179	T
	FASTmrEMMA	chrom_06_9260283	6	9,260,283	8.5826	9.4891	7.4136	0.2572	C
	pLARmEB	chrom_09_6543874	9	6,543,874	6.3538	7.1985	Na	0.3468	C
		chrom_19_9509263	19	9,509,263	6.9294	7.7918	Na	0.1243	T
		chrom_01_13919773	1	13,919,773	8.33	9.2304	Na	0.4737	T
		chrom_09_12686050	9	12,686,050	6.4971	7.3464	Na	0.2982	A
		chrom_20_24056604	20	24,056,604	11.0955	12.0555	Na	0.3158	T
		chrom_20_3216465	20	3,216,465	6.2132	7.0533	Na	0.2398	C
	ISIS EM-BLASSO	chrom_06_9260283	6	9,260,283	9.7758	10.7094	Na	0.2572	C
		chrom_14_4221541	14	4,221,541	6.1297	6.9671	Na	0.1156	G
		chrom_20_24056604	20	24,056,604	6.9764	7.8402	Na	0.3179	T
K2 + Q	mrMLM	chrom_02_19256555	2	19,256,555	7.2774	8.1499	Na	0.1374	C
		chrom_08_4795920	8	4,795,920	7.08	7.9469	Na	0.1053	T
		chrom_09_29476318	9	29,476,318	7.5647	8.4452	5.7964	0.2895	T
		chrom_12_4768042	12	4,768,042	6.4595	7.3076		0.1345	A
	FASTmrMLM	chrom_11_11707748	11	11,707,748	7.7098	8.5942		0.4335	T
		chrom_14_4221541	14	4,221,541	7.5882	8.4693		0.1156	G
		chrom_15_18362333	15	18,362,333	6.3664	7.2115		0.3526	T
		chrom_19_9509263	19	9,509,263	13.4956	14.4966		0.1243	T
	FASTmrEMMA	chrom_20_3216465	20	3,216,465	10.5269	11.4758	10.3658	0.237	C
	pLARmEB	chrom_09_6543874	9	6,543,874	6.3538	7.1985	Na	0.3468	C
		chrom_19_9509263	19	9,509,263	6.9294	7.7918	Na	0.1243	T
		chrom_01_13919773	1	13,919,773	6.8541	7.7143	Na	0.4737	T
		chrom_09_12686050	9	12,686,050	8.2327	9.1306	Na	0.2982	A
		chrom_20_24056604	20	24,056,604	11.2556	12.2186	Na	0.3158	T
		chrom_20_3216465	20	3,216,465	6.0554	6.8903	Na	0.2398	C
	ISIS EM-BLASSO	chrom_06_9260283	6	9,260,283	9.7758	10.7094	Na	0.2572	C
		chrom_14_4221541	14	4,221,541	6.1297	6.9671	Na	0.1156	G
		chrom_20_24056604	20	24,056,604	6.9764	7.8402	Na	0.3179	T
K3 + Q	mrMLM	chrom_03_15679299	3	15,679,299	8.6532	9.5614	Na	0.1725	C
	mrMLM	chrom_03_3532422	3	3,532,422	6.3269	7.1707	Na	0.2281	A
	mrMLM	chrom_08_5003338	8	5,003,338	6.8525	7.7127	2.1301	0.2895	A
	mrMLM	chrom_09_6543874	9	6,543,874	6.0707	6.9061	Na	0.345	C
	mrMLM	chrom_10_23363999	10	23,363,999	7.851	8.7391	Na	0.1374	T
	FASTmrMLM	chrom_06_9260283	6	9,260,283	6.3418	7.1861	Na	0.2572	C
	FASTmrMLM	chrom_09_6543874	9	6,543,874	7.1057	7.9733	Na	0.3468	C
	FASTmrMLM	chrom_11_11707748	11	11,707,748	7.9179	8.8078	Na	0.4335	T
	FASTmrMLM	chrom_14_4221541	14	4,221,541	8.9017	9.8158	Na	0.1156	G
	FASTmrMLM	chrom_19_9509263	19	9,509,263	9.1536	10.0735	Na	0.1243	T
	FASTmrMLM	chrom_20_23996201	20	23,996,201	6.3599	7.2048	Na	0.2948	C
	pLARmEB	chrom_09_6543874	9	6,543,874	6.3538	7.1985		0.3468	C
	pLARmEB	chrom_19_9509263	19	9,509,263	6.9294	7.7918		0.1243	T
	pKWmEB	chrom_17_13117447	17	13,117,447	7.0769	7.9437	0.3538	0.2456	A
	pKWmEB	chrom_20_24056604	20	24,056,604	8.7266	9.6366		0.3158	T
	pKWmEB	chrom_20_3216465	20	3,216,465	7.8566	8.7449		0.2398	C
	pKWmEB	chrom_20_3389269	20	3,389,269	7.8727	8.7614		0.3421	C
	ISIS EM-BLASSO	chrom_06_9260283	6	9,260,283	9.7758	10.7094		0.2572	C
	ISIS EM-BLASSO	chrom_14_4221541	14	4,221,541	6.1297	6.9671		0.1156	G
K4 + Q	ISIS EM-BLASSO	chrom_20_24056604	20	24,056,604	6.9764	7.8402		0.3179	T
	mrMLM	chrom_08_5003338	8	5,003,338	6.5056	7.3551	2.4659	0.2895	A
	mrMLM	chrom_09_6543874	9	6,543,874	8.7343	9.6445	1.75	0.345	C
	mrMLM	chrom_18_11140965	18	11,140,965	7.536	8.4157	1.7134	0.2661	G
	FASTmrMLM	chrom_06_9260283	6	9,260,283	6.3418	7.1861	2.12	0.2572	C
	FASTmrMLM	chrom_09_6543874	9	6,543,874	7.1057	7.9733	11.2	0.3468	C
	FASTmrMLM	chrom_11_11707748	11	11,707,748	7.9179	8.8078	2.45	0.4335	T
	FASTmrMLM	chrom_14_4221541	14	4,221,541	8.9017	9.8158	1.34	0.1156	G
	FASTmrMLM	chrom_19_9509263	19	9,509,263	9.1536	10.0735	1.26	0.1243	T
	FASTmrMLM	chrom_20_23996201	20	23,996,201	6.3599	7.2048	1.09	0.2948	C
	pLARmEB	chrom_09_6543874	9	6,543,874	6.3538	7.1985	6.27	0.3468	C
	pLARmEB	chrom_19_9509263	19	9,509,263	6.9294	7.7918	12.23	0.1243	T
	pKWmEB	chrom_17_13117447	17	13,117,447	7.0769	7.9437	8.23	0.2456	A
	pKWmEB	chrom_20_24056604	20	24,056,604	8.7266	9.6366	3.56	0.3158	T
	pKWmEB	chrom_20_3216465	20	3,216,465	7.8566	8.7449	7.17	0.2398	C
	pKWmEB	chrom_20_3389269	20	3,389,269	7.8727	8.7614	8,90	0.3421	C
	ISIS EM-BLASSO	chrom_06_9260283	6	9,260,283	9.7758	10.7094	2.12	0.2572	C
	ISIS EM-BLASSO	chrom_14_4221541	14	4,221,541	6.1297	6.9671	1.56	0.1156	G
	ISIS EM-BLASSO	chrom_20_24056604	20	24,056,604	6.9764	7.8402	3.6	0.3179	T

- KQ Model with Different Kinship Matrices.

We then applied the KQ model with kinship matrices, incorporating varying levels of markers with different PICs alongside the population structure as covariates.

**Fig. 5:**
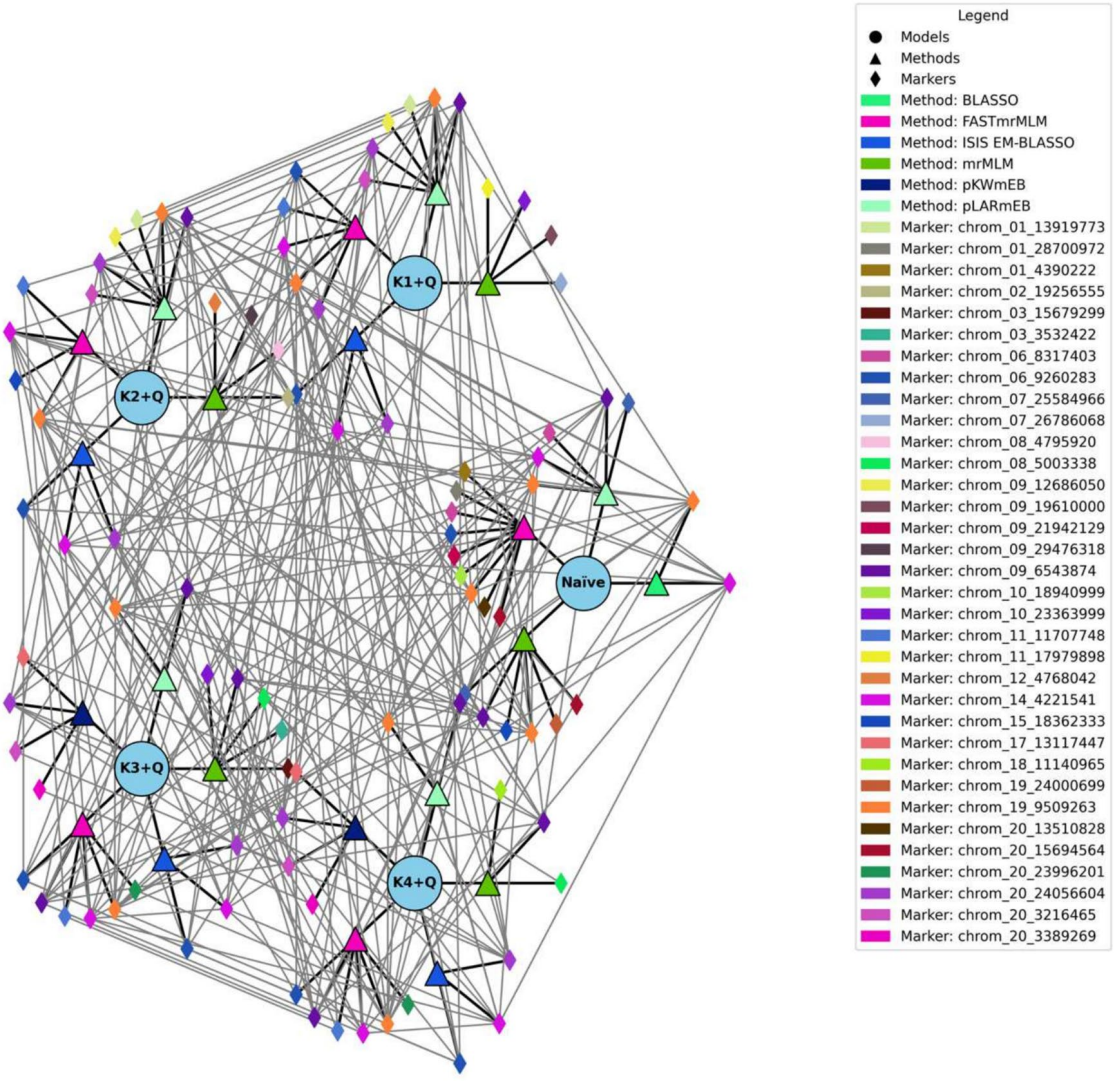
Network Analysis displaying common markers across the six genetic models.

- Using a kinship matrix for markers with low PIC below 0.1, we identified 13 unique SNP markers spread across 9 yam chromosomes. All six methods consistently detected these markers. However, a significant discrepancy was observed between the expected and observed log-likelihood values (Fig. 4).
- When the kinship matrix included markers with PIC between 0.1 and 0.2, 14 unique SNPs were identified across 11 chromosomes. These yield-linked markers displayed high LOD scores but also exhibited a substantial inflation between observed and expected LOD values.
- Finally, incorporating the kinship matrix originating from high PIC as a covariate in the KQ model analysis revealed 12 unique markers associated with tuber yield (Table 2). These markers had high MAF and explained a significant portion of the phenotypic variance. Interestingly, the markers identified using a high heterozygosity kinship matrix showed a strong correlation between the expected and observed LOD values (Fig. 4). It is worth noting that several markers were identified across the six genetic models (Fig. 5).

### Markers effect and yield prediction

**Fig. 6:**
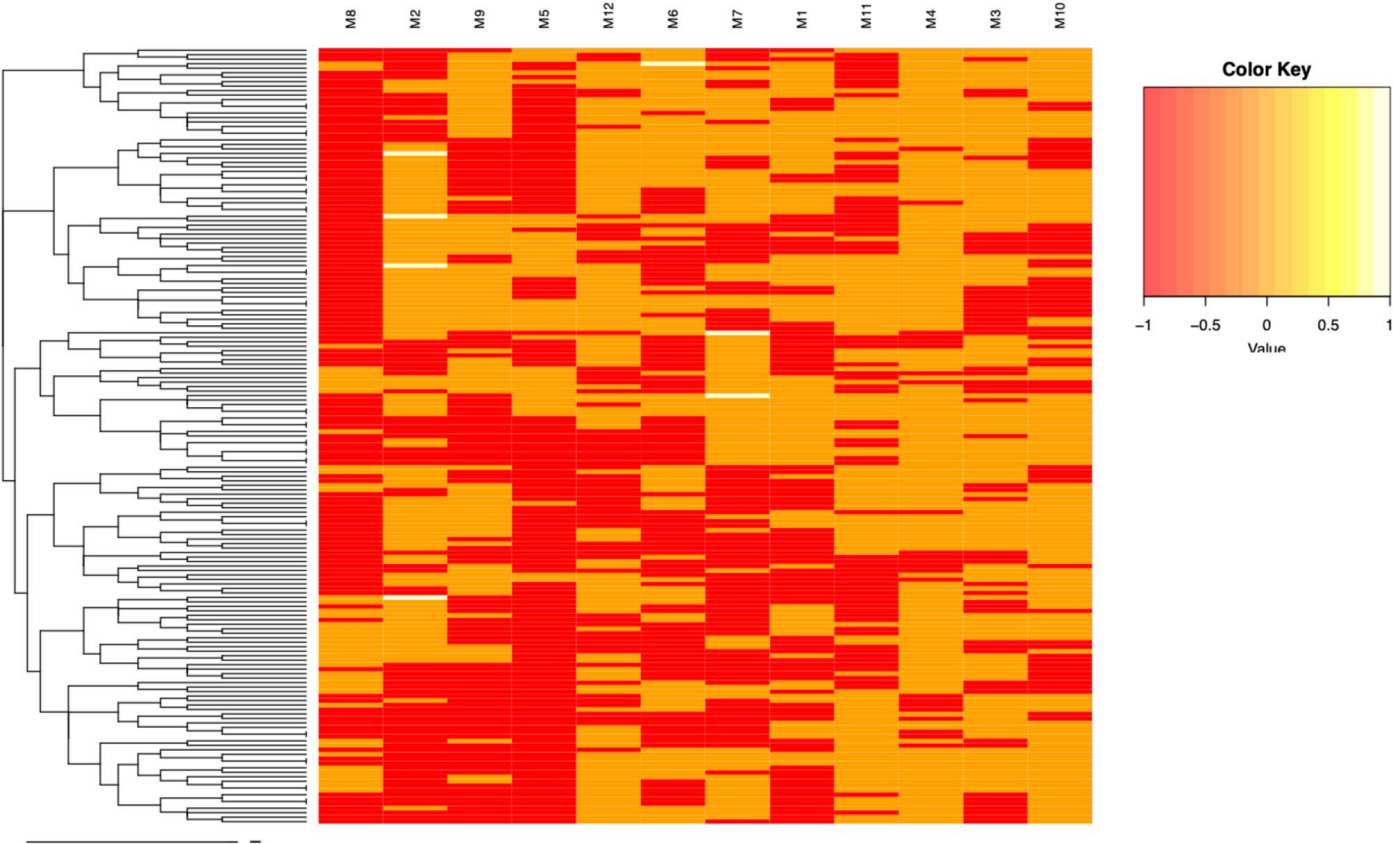
Heatmap displaying the effect of the 12 markers associated with the tuber yield of white yam identified through the K4 + Q model.

**Fig. 7:**
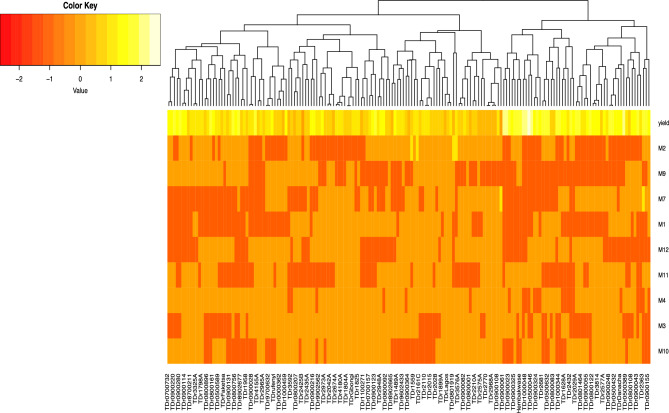
Heatmap displaying the effect of the nine discriminant SNP markers associated with the tuber yield of white yam identified through the K4 + Q model.

For this only model K4 + Q model was used for the yield prediction. Our analysis identified three markers (M5, M8, and M9) from the K4 + Q model as non-informative for predicting yield across all yam genotypes (Fig. 6). The remaining nine markers displayed high segregation classifying the 173 yam genotypes into high-yielding and low-yield (Fig. 7). Analysis of the 9 SNP markers revealed two distinct clusters among the evaluated yam genotypes. These clusters corresponded to low-yielding and high-yielding genotypes, as indicated by the color key. Interestingly, the high-yielding cluster was predominantly composed of elite breeding clones.

### Gene annotation and putative gene function

Using the K4 + Q model and annotation analysis, we identified ten genes in Table 3 potentially linked to tuber yield. These genes include those related to protein kinase activity (IPR000719, IPR011009, IPR002290, IPR017441) and Leucine-rich repeat domains (IPR006553). Interestingly, genes associated with the Brevis and Transcription factor BREVIS RADIX, N-terminal domain (IPR027988) were also found on chromosome 20 (Table 3).

**Table 3:** List of putative genes associated with tuber yield with their respective function.

Markers	Chr	Gene Pos	Gene ID	Putative gene/enzyme	Functions
chrom_06_9260283	6	9,250,888	IPR000719	Protein kinase domain	Enhancing the tolerance of plant to drought and water stress thereby improving the yield
chrom_08_5003338	8	5,000,118	IPR011009	Protein kinase-like domain	
chrom_09_5003338	9	5,001,008	IPR006553	Leucine-rich repeat, cysteine-containing subtype	It confers resistance virus in potato thereby enhancing tuber development and yield
chrom_11_11707748	11	11,705,276	IPR002290	Serine/threonine- / dual specificity protein kinase, catalytic domain	- Participate in the process of starch and sugar biosynthesis in potatoes and stimulated glucose pyrophosphorylase - Stimulate some enzymes in the starch biosynthesis pathways in potato and wheat
chrom_14_4221541	14	4,221,005	IPR017441	Protein kinase, ATP binding site	Enhancing the tolerance of potato plant to drought and water stress thereby improving the yield
chrom_19_9509263	19	9,508,211	IPR0135591	Brevis radix (BRX) domain	It regulates cell proliferation and elongation in the root It involves in root growth in Arabidopsis
chrom_20_24056604	20	24,052,045	IPR027988	Transcriptional factor BREVIS RADIX, N-terminal dormain	Characterized as being a transcription factor in plants regulating the extent of cell proliferation and elongation in the growth zone of the root Involved in cytokinin-mediated inhibition of lateral root initiation in Arabidopsis Promotes shoot growth in Arabidopsis

## Discussion

In this study, we employed six different genetic models in the multi-random mixed linear (mrMLM) GWAS model to identify SNPs that were significantly associated with tuber yield per plant in White Guinea yam, considering various levels of heterozygosity.

### Phenotypic yield variation and population structure

The observed variation in yield among the yam genotypes highlights the potential for genetic improvement through breeding programs. The population structure analysis through the admixture and clustering revealed the presence of three well-distinct clusters, suggesting the presence of genetic variation that can be exploited for breeding purposes. Traits association analysis relies on a population panel with a high diversity. Similar trending results were obtained in the previous study conducted on trait association by^15,19,24^.

### Marker polymorphism and trait association

We performed trait association analyses by evaluating different levels of marker polymorphism using a range of genetic models from the naïve (uncontrolled) model to the more robust K (kinship) + Q (population structure) model. Our results revealed that the influence of population structure on marker-trait associations was strongly affected by the level of polymorphism, as reflected by the Polymorphism Information Content (PIC). Markers with higher PIC values tend to capture greater allelic diversity, thereby increasing the potential to detect rare alleles within genetically heterogeneous populations. Such alleles, although infrequent, can have significant effects on complex traits and are particularly valuable in breeding programs aiming to exploit novel genetic variation. As demonstrated in other yam studies by Shental et al.^25^ and Cormier et al.^26^, rare alleles identified through high-PIC markers are often associated with important agronomic traits, including yield components and stress adaptation.

In yam, several GWAS efforts have successfully identified quantitative trait loci (QTL) linked to tuber yield and related traits. For instance, Cormier et al.^26^) identified yield-associated loci in *D. alata* using GBS-based SNPs, while Bredeson et al.^27^ highlighted structural variation contributing to tuber size and shape. Similarly, Shenta et al.^25^ emphasized the role of population structure and heterozygosity in trait discovery, suggesting that careful control for these factors is essential for accurate association mapping. In our study, multiple SNP markers were significantly associated with tuber yield per plant under both naïve and controlled models. Among the tested models, the K + Q model showed the best fit, as illustrated by the Q-Q plots, which indicated improved control of false positives and a closer alignment between observed and expected LOD scores.

The benefits of maintaining heterozygosity in yam populations are particularly significant in the context of GWAS. As highlighted by Jia et al.^28^ and supported by our findings, high heterozygosity enhances the power of association studies by providing a broader range of alleles for analysis. This is crucial for dissecting the polygenic nature of yield and for uncovering QTLs with both large and small effects. The SNP markers identified in this study as significantly associated with tuber yield are promising candidates for marker-assisted selection (MAS) and genomic prediction efforts. They offer valuable targets for breeding programs focused on improving productivity in *D. rotundata*, especially under diverse environmental conditions.

### Gene annotation and candidate genes

Functional annotation of the identified SNP markers revealed genes potentially involved in various processes influencing yield, such as starch biosynthesis, nutrient uptake, and stress tolerance^29,30^. Functional annotation of these genes can illuminate the biological pathways underlying yield variation in yam. By effectively addressing the challenge of high heterozygosity, yam breeding can leverage GWAS to identify genes that control desirable traits such as yield; this will ultimately lead to the development of superior yam varieties, contributing to enhanced food security and improved livelihoods for yam farmers. Further validation through functional studies is necessary to confirm the role of these candidate genes in yam yield.

### Conclusion and perspective

Using six genetic models (Multi-locus random-SNP-effect Mixed Linear Model, Fast multi-locus random-SNP-effect EMMA (FASTmrEMMA), Iterative Sure Independence Screening EM-Bayesian LASSO (ISIS EM-BLASSO), Polygenic-background-control-based Least Angle Regression plus Empirical Bayes (pLARmEB), Polygenic-background-control-based Kruskal–Wallis test plus Empirical Bayes (pKWmEB), Fast mrMLM (FASTmrMLM) with varying levels of PIC, we assessed the impact of PIC on population structure and its implications for trait association in yam. Several markers significantly associated with tuber yield per plant were identified in White Guinea yam (*Dioscorea rotundata*). Annotation analysis of these markers revealed multiple putative genes with diverse functions related to plant growth and biomass accumulation. The functional validation of these candidate genes through approaches such as gene expression profiling or gene editing will be essential to confirm their roles and strengthen the association between the identified SNPs and yield. Overall, this study advances our understanding of the genetic architecture underlying yield in yam and provides a solid foundation for developing trait-linked markers to support marker-assisted selection (MAS). By integrating population structure analysis, genetic diversity assessment, and GWAS, we highlight the critical role of PIC in improving complex traits like yield, paving the way for more efficient and targeted yam breeding strategies.

## Materials and methods

### Genetic material and plant phenotyping

A comprehensive study was conducted using 173 genotypes of *D. rotundata*, comprising 86 elite breeding clones, 77 genebank accessions, and ten farmers’ varieties. The detailed information regarding these genotypes has been previously documented by Darkwa et al.^31^. Over a span of 2 years, field experiments were carried for two consecutive years 2017–2018 and 2018–2019 at the International Institute of Tropical Agriculture (IITA/Ibadan Nigeria), situated at an altitude of 221 m and coordinates 07° 29.639″ N, 003° 54.092″ E. The experiment employed augmented row–column designs for the respective years. In the first year, 43 accessions were replicated four times, with the remaining accessions non-replicated in single-row plots consisting of two plants, spaced at one-meter intervals within rows, in plot areas of 2 m^2^. In the subsequent year, the trial utilized a single plant per plot arranged in a randomized block design, replicated three times. The experimental field was meticulously maintained weed-free through manual weed control throughout the growth phases of the plants. The phenotypic assessment of the accessions was focused on fresh root yield per plant at harvest, employing the yam ontology framework described by Asfaw^32^.

A two-stage analysis was conducted to generate the Best Linear Unbiased Estimates (BLUEs) for the GWAS using mixed linear model in Lme4 R package^33^ accounting for the different experimental designs used for each of the single trials.

The model used for the randomized block design: ${Y}_{ik}=\mu + {G}_{i}+ {{R}_{j}+Row}_{k}+{Col}_{l}+ {\varepsilon }_{ikl}$

Where ${Y}_{ik}$ = Phenotypic value, $\mu$ = grand mean, ${G}_{i}$ = effect of the *i*th genotype, ${R}_{k}$= effect of the *k*th replication, ${\varepsilon }_{ik}$ = residual.

For the augmented design, the model was fitted by replacing the ${R}_{k}$ with the ${B}_{j}$ as the block effect in the following model:

$${Y}_{ik}=\mu + {G}_{i}+ {B}_{j} {+ Row}_{k}+{Col}_{l}+ {\varepsilon }_{ikl}$$

The genotype was considered as fixed effect in the models and the best linear unbiased estimates (BLUES) were generated. The genotype was first fitted as a random effect in each of the models to estimate the heritability of tuber yield per plant according to the formula described by Cullis et al.^34^.

$${H}_{Cullis}^{2}=1-\frac{{V}_{\Delta }^{BLUP}}{2{\sigma }_{g}^{2}}$$

where ${V}_{\Delta }^{BLUP}$ is the average standard error of the clonal genotypic BLUPs (best linear unbiased predictions) and ${\sigma }_{g}^{2}$, is the clonal genotypic variance.

A linear mixed model was fitted in the second stage analysis as:

$${y}_{ijk}=\mu + {G}_{i}+ {Y}_{j} + {(GY)}_{ij}+\upepsilon$$

where ${y}_{ijk}$ is the adjusted means (BLUEs) of the *i*th genotype in year *j* obtained from the linear mixed model in stage 1, $\mu$ = grand mean, ${G}_{i}$ = main effect of the *i*th genotype, ${Y}_{j}$ = main effect of the *j*th year, ${(GY)}_{ij}$ = is the genotype *i* in year *j* interaction effect, $\upepsilon$ = the error associated with the estimation the adjusted means with variance matrix assumed known from stage 1. The genotype was considered as fixed effect and a weighted-combined mixed model fitted using BLUES and weights obtained from the first stage analysis to obtain the adjusted means of each genotype^35^.

### DNA extraction and SNP calling

DNA extraction and SNP calling processes were conducted at the Iwate Biotechnology Research Center (IBRC-Japan). Lyophilized leaves were sent for DNA extraction, library construction, and whole-genome resequencing. Total genomic DNA was extracted from the leaf samples using a NucleoSpin Plant II Kit following the manufacturer’s protocol (MachereyNagel GmbH & Co), with minor adjustments.

The generated paired-end sequencing reads in fastq format were aligned to the *D. rotundata* reference genome version 2 using Hisat2. SAM files were converted to BAM format and sorted by name using SAMtools. In cases where multiple sequencing samples originated from the same biological clone, the sorted BAM files for each clone were merged using SAMtools. Duplicate reads were identified and read groups were added using the Picard package (v2.17.5).

GATK (v3.8–0) was employed for indel realignment, variant calling (HaplotypeCaller in gVCF mode), and joint genotyping (GenotypeGVCFs). The resulting VCF file was filtered based on minor allele frequency (MAF > 0.1) and absence of missing data at the genotype and SNP marker levels. Only bi-allelic SNP markers with a genotype quality > 20 and a read depth > 5 were retained post-filtering using vcftools and plink. Subsequently, the SNPs underwent linkage disequilibrium (LD) pruning (LD pruning was conducted with PLINK using –indep-pairwise 50 5 0.5 to reduce redundancy) with parameters including a window size of 50 bp in SNPs, a step size of 5 for shifting the window, an R-square value of 0.5, resulting in the retention of 136,429 SNP markers for all subsequent analyses.

### Genetic diversity and population structure analysis

Various population genetic analysis methods were conducted to explore the structure and level of genetic diversity in the study material. The SNP distribution and the density were estimated using the ‘Cmplot’ function implemented in the CMplot R package^36^. Summary statistics such as the minor allele frequency (MAF), the observed heterozygosity (H), and the polymorphism information content (PIC) were estimated using the function "–freq" and "–hardy" using PLINK V1.90^37^. To understand the relationship among and between genebank accession, farmers varieties and elite breeding clones, we conducted diversity analysis using phangorn library package,.

The Structure software version 2.3.3 was used to cluster samples into populations. Structure simulations were conducted using an admixture model with a burn-in period of 20,000 iterations and a Markov chain Monte Carlo (MCMC) set at 20,000 iterations. The simulations were repeated three times for K-values ranging from 1 to 10. The optimal subpopulation model was determined by applying the informal pointers (e.g., geographical origin) proposed by Pritchard et al.^38^ and Falush et al.^39^ considering ΔK, a second-order rate change with respect to K, as defined by Evanno et al.^40^ and implemented in STRUCTURE HARVESTER. The most likely value of K was then determined, and the Structure population was plotted using the barplot function in R. The phylogenetic tree was constructed using the ape version 5.0 package in R.

### Genome-wide association study

Genome-wide association studies (GWAS) were conducted using six multi-locus models implemented in the R package mrMLM v4.0.2^41^. These models included:

- Multi-locus random-SNP-effect Mixed Linear Model^42^
- Fast multi-locus random-SNP-effect EMMA (FASTmrEMMA)^43^
- Iterative Sure Independence Screening EM-Bayesian LASSO (ISIS EM-BLASSO)^44^
- Polygenic-background-control-based Least Angle Regression plus Empirical Bayes (pLARmEB)^41^
- Polygenic-background-control-based Kruskal–Wallis test plus Empirical Bayes (pKWmEB)^43^
- Fast mrMLM (FASTmrMLM)^44^

**Fig. 8:**
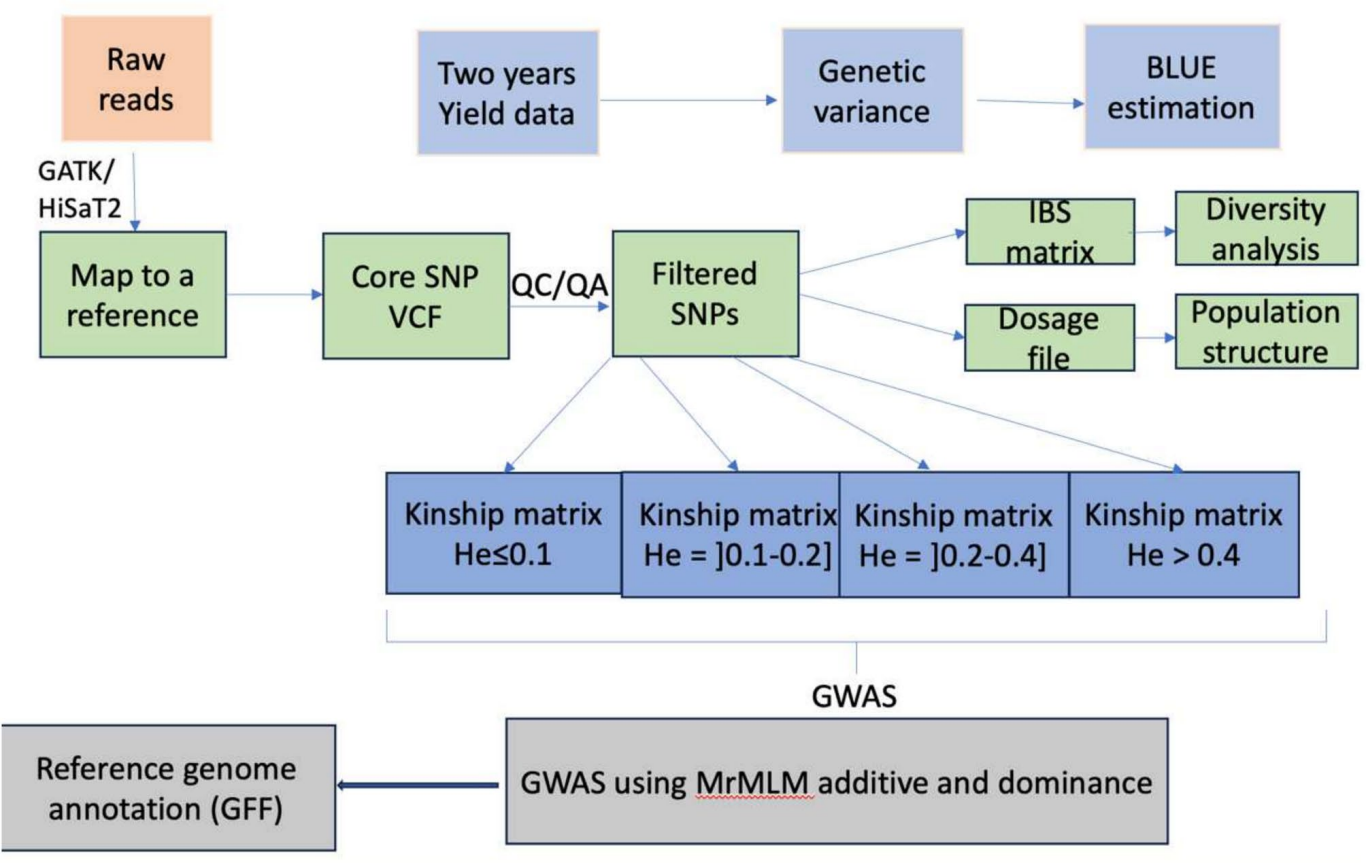
SNP Filtering and graphical display of GWAS approach.

To account for false positive discovery, we incorporated a population structure matrix (Q) and different kinship matrix generated based on different markers heterozygosity levels (Fig. 8). The Kinship matrices were generated using the VanRaden method in GAPIT. The mrMLM package (v4.0.2) was used to estimate the percentage of phenotypic variation explained by each associated marker (R^2^) and their corresponding effects (https://cran.r-project.org/web/packages/mrMLM/index.html).

### Gene annotation and putative gene prediction

Putative genes closely linked to the trait markers were investigated using the GFF3 file generated from the yam reference Genome v2^10^. The genes were searched in the region of 500Kbp, and their respective function was documented using the NCBI and INTERPRO websites for gene ontology documentation. To validate the maker’s ability to predict the tuber yield as an in-vivo method, we considered all markers as factors, and a heatmap was defined, while the marker effect was detected based on color gradient.

## Supplementary Information

Supplementary Information.

## Data Availability

The datasets generated and analysed during the current study are available in the Figshare repository, through the following link https://figshare.com/articles/dataset/Phenotypic_data/27169110
