# Supplementary material for: Genome wide association studies in yam reveal the challenge of high heterozygosity
Source: Sci Rep. 2025 Jul 24;15:26929. doi: 10.1038/s41598-025-10344-z (PMC12289929; doi:10.1038/s41598-025-10344-z)
Supplement: Supplementary file 1 — Supplementary Information. [file 41598_2025_10344_MOESM1_ESM.docx]

Supplementary Figure 1. Yam yield per plant distribution


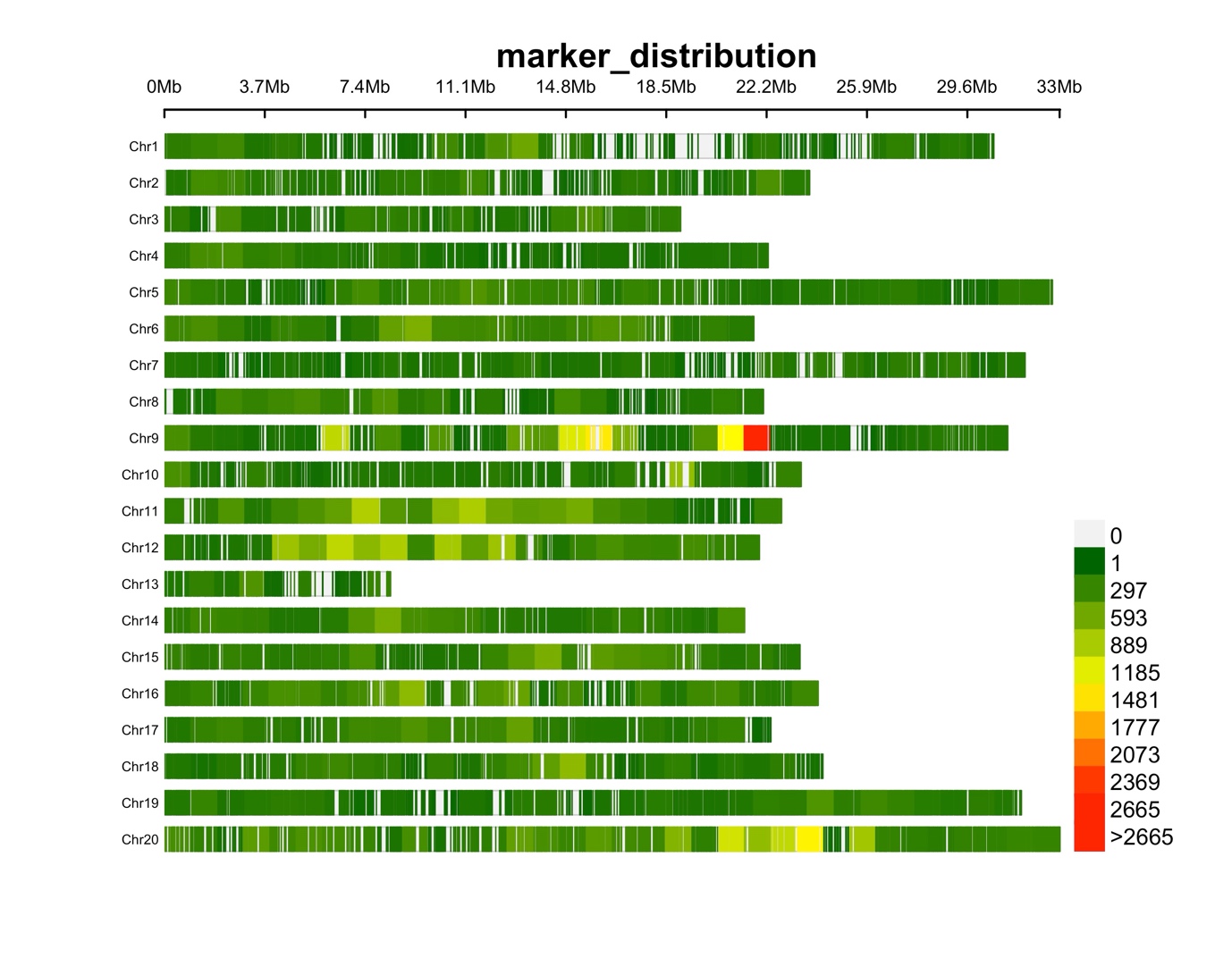


Supplementary Figure 2. SNP Markers distribution and density across the 20 yam chromosomes
